# Staffing and leadership as drivers of nurse retention: A work environment analysis

**DOI:** 10.1097/nmg.0000000000000343

**Published:** 2026-02-03

**Authors:** Lyn Stankiewicz Losty, Angela Pascale, Nora E. Warshawsky

**Affiliations:** **Lyn Stankiewicz Losty** is Contributing Faculty at Walden University in Corolla, SC. At Press Ganey Associates LLC in South Bend, IN, **Angela Pascale** is a Research Analyst and **Nora E. Warshawsky** is a Nurse Scientist.

**Keywords:** missed nursing care, nurse outcome, nurse retention, nurse work environment, practice environment scale, staffing and resource adequacy

## Abstract

**Background:**

Nurse work environment (NWE) is a well-established predictor of nurse and patient outcomes. However, limited research has examined the individual contributions of NWE domains to nurse outcomes.

**Purpose:**

This study aimed to identify which subscales of the Practice Environment Scale (PES) most strongly influence nurse outcomes: retention, job enjoyment, respect and recognition, and missed nursing care.

**Methods:**

A cross-sectional analysis was conducted using 2024 National Database of Nursing Quality Indicators® RN Survey data from 1789 inpatient units across 221 hospitals. NWE was measured using PES subscales, and nurse outcomes were assessed via standardized instruments. Structural equation modeling evaluated direct effects of PES domains on outcomes.

**Results:**

All PES subscales were significantly associated with nurse outcomes (*P* < .05). Staffing and resource adequacy had the strongest effects on retention (β = 0.44), job enjoyment (β = 0.61), and reduced missed care (β = -0.87). Nurse manager leadership and support also significantly predicted retention and respect and recognition. Nurse participation in hospital affairs primarily influenced respect and recognition, whereas nurse-physician interaction showed weaker associations.

**Conclusions:**

Adequate staffing and strong nurse manager leadership are critical to nurse retention and satisfaction. Targeted improvements in these domains may enhance workforce stability and care quality.

**Figure:**
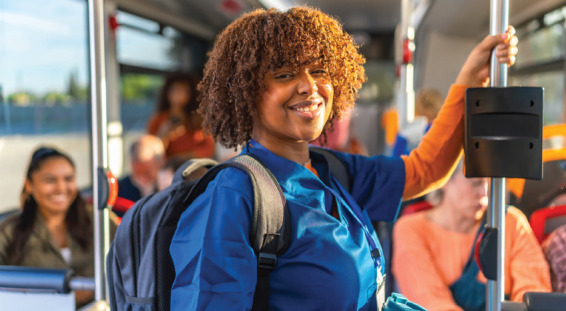
No caption available.

Nurse managers are instrumental in achieving patient, provider, and organizational outcomes.^1^ Managing a novice workforce, navigating new care delivery models, prioritizing health and well-being among nurses, and addressing concerns about workplace violence are common challenges that nurse managers face.^2^ They must develop tactics to recruit, retain, and lead the next generation of nurses through these challenges given their role as administrative leaders on their units.^3^ The expectations of nurse managers are growing as health care organizations face greater financial burdens with many underprepared to serve in their leadership roles.

A significant stressor among nurse managers is the challenge of matching adequate staffing to patient care needs while conserving expenses.^4^ Although staffing has been a long-standing challenge, it has steadily grown in complexity since the COVID-19 pandemic. During this time, the number of experienced baby boomer nurses have decreased due to retirements as younger Generation Z (Gen Z) nurses began entering the profession, creating an “experience-complexity gap.”^5^ Meeting the developmental needs of the nursing workforce is just one part of the equation. Gen Z nurses' changing values toward work are reflected in their reluctance to work full-time and off-shifts.^5^ The increase in part-time nurses means higher numbers of nurses are reporting to individual nurse managers. Further complicating nurse staffing are newer models of care delivery that add licensed practical nurses and virtual nurses into the mix, resulting in increased stress and burnout among nurse managers.^3^ It is imperative that nurse managers implement strategies to mitigate the burdens of their unit workforce.

Fortunately, nursing has a strong foundation of evidence demonstrating the value of the nurse work environment (NWE). The NWE has stood the test of time as a protective factor against internal and external challenges nurse managers face. Extensive evidence demonstrates that high-quality NWE are associated with better nurse outcomes and quality patient care.^6^,^7^ Key outcomes associated with high-quality NWEs include fewer adverse safety events and lower levels of lower burnout, higher job satisfaction, and improved retention among nurses.^7^ Given the importance of the NWE in driving positive nurse outcomes, understanding the dimensions of a positive practice environment is a critical starting point for developing strategies to recruit and retain nurses.

The NWE is most commonly assessed using the Practice Environment Scale of the Nursing Work Index (PES-NWI) developed by Lake.^8^ Lake defined the NWE as “the organizational characteristics of a work setting that facilitate or constrain professional nursing practice.”^8^ The PES is conceptualized as five domains of the NWE that enhance or attenuate a nurse's ability to deliver high-quality nursing care.^8^ Few studies have evaluated the impact of the five subscales individually on nurse outcomes.^9^ Thus, the purpose of this study was to understand the relationship between the individual PES subscales and key nurse outcomes to identify strategies to recruit and retain nurses.

## METHODS

### Study design and sample

This cross-sectional study examined the influence of PES domains on nurse outcomes using inpatient unit-level data from the annual 2024 National Database of Nursing Quality Indicators® (NDNQI®) RN Survey. Press Ganey client hospitals voluntarily participate in the annual RN survey to collect data on nurse outcomes, new, and individual characteristics of registered nurses (RNs). The NDNQI RN Survey is distributed to eligible RNs who have been employed for a minimum of 3 months in their current unit and spend at least 50% of their time providing direct patient care. The Advarra Institutional Review Board (IRB) reviewed the NDNQI data management procedures and deemed the study exempt from IRB oversight. The study sample consisted of 1789 inpatient units across 221 hospitals with complete data on PES subscales and nurse outcomes.

### Measures

#### Practice Environment Scale (PES)

NWE was measured using the PES.^8^ The PES assesses nurse perceptions of their work environment with 31 items across five subscales: Nurse Participation in Hospital Affairs; Nursing Foundations for Quality of Care; Nurse Manager Ability, Leadership and Support of Nurses; Staffing and Resource Adequacy; and Collegial Nurse-Physician Relations. Each item is rated on a 4-point Likert-type scale ranging from 1 (strongly disagree) to 4 (strongly agree).

#### Nurse outcomes

**Missed nursing care**. Missed nursing care is the unit-level average of important activities that nurses report were left undone due to time constraints. Participants are asked “Which of the following activities were necessary but left undone because of time constraints?”, and are instructed to check all applicable items from a list. Sample response options included: adequately document nursing care, treatments and procedures, coordinate patient care, and administer medications on time. Each nurse reported the total number of nursing activities missed during their last shift, with a possible of 0 to 15 missed nursing activities. For the current study, missed nursing care was calculated as the average unit-level score of the total reported missed care events among nurses on that unit during their last shift.

**Retention**. Retention was measured as the percentage of nurses who plan to remain in direct patient care on their current unit for the next year.

**Job enjoyment**. Job enjoyment was measured using the composite score of the 5-item Job Enjoyment Scale in the NDNQI RN Survey. This scale assesses nurses' satisfaction with their current role on a 6-point scale ranging from 1 (strongly disagree) to 6 (strongly agree).

**Respect and recognition**. Respect and recognition were measured using the composite score of the 4-item Eyes of Workforce scale that measures nurses' perceptions of being treated with respect and recognized within their organization on a 5-point scale ranging from 1 (never) to 5 (every day).

### Data analysis

Simple linear regression models were used initially to independently assess the association between each of the five PES subscales and each of the four nurse outcomes to understand how each individual NWE domain related to each outcome. Then, structural equation modeling was conducted using the lavaan package in R to test a comprehensive path model. Direct paths were specified from each PES subscale to all four nurse outcomes. Additionally, correlations among the PES subscales were freely estimated to account for conceptual overlap and high intercorrelations, allowing each subscale's unique contribution to nurse outcomes to be assessed while controlling for shared variance. The model was fully saturated with zero degrees of freedom, due to the inclusion of all direct paths and predictor covariances. As a result, traditional fit indices weren't interpreted, and model evaluation was focused on the estimation of standardized path coefficients and the proportion of the variance explained (R^2^) in each outcome. Statistical significance was evaluated at the 0.05 level.

## RESULTS

Hospital and unit characteristics of the sample are presented in Table 1 and descriptive statistics of the PES subscales and nurse outcome variables are presented in Table 2. All simple linear regression models examining independent associations between each PES subscale and each nurse outcome were statistically significant (*P* < .05) indicating that each individual NWE domain had a measurable relationship with each of the four nurse outcomes.

**TABLE 1:** Unit and hospital characteristics

	n	%
**Hospital characteristics (N = 221 hospitals)**		
***Bed size***		
<100	78	35.29
100-199	55	24.89
200-299	46	20.81
300-399	22	9.95
400-499	6	2.71
≥500	14	6.33
***Magnet status***		
Magnet	65	29.41
Non-Magnet	156	70.59
***Teaching status***		
Academic medical center	11	4.98
Nonteaching	141	63.80
Teaching	69	31.22
**Unit-level characteristics (N = 1789 units)**		
Adult inpatient	1323	73.95
Obstetric inpatient	229	12.80
Neonatal inpatient	100	5.59
Pediatric inpatient	78	4.35
Rehab inpatient	59	3.30

**TABLE 2:** Descriptive statistics of study sample (N = 1789 units)

	Mean	SD	Min	Max
**PES subscales**				
Staffing and Resources	2.79	0.39	1.30	3.96
Nurse Manager Leadership and Support	3.16	0.31	1.77	3.98
Foundations for Quality of Care	3.18	0.21	2.43	3.98
Nurse Participation	2.97	0.28	1.49	3.97
Nurse-Physician Interaction	3.13	0.27	2.06	3.99
**Nurse outcomes**				
Intent to stay (%)	74.54	16.30	0.00	100.00
Missed nursing care	2.00	1.10	0.00	8.11
Job enjoyment	4.07	0.54	2.22	5.90
Respect and recognition	4.07	0.32	2.17	5.00

**Key:** PES, Practice Environment Scale

**FIGURE 1:**
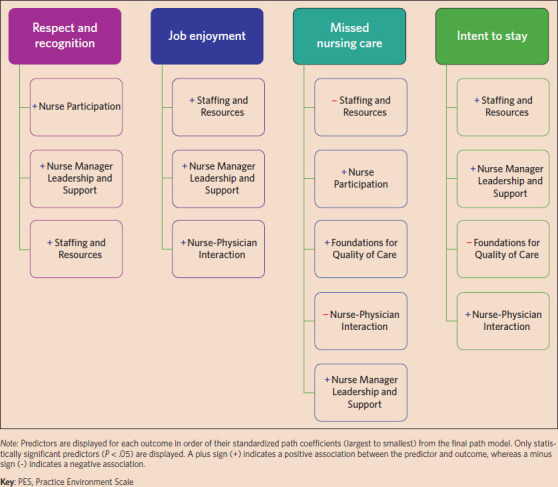
Graphic representation of significant PES subscale predictors of nurse outcomes from the path model, ordered by effect size

Both Table 3 and Figure 1 present the results from the structural equation model evaluating the direct effects of the five PES subscales on the four nurse outcomes. Staffing and resource adequacy demonstrated the strongest and most consistent associations with the outcomes. It was significantly associated with greater retention (β = 0.44), greater job enjoyment (β = 0.61), higher respect and recognition (β = 0.24), and lower missed nursing care (β = -0.87). This domain had the strongest effect on job enjoyment, retention, and missed nursing care.

**TABLE 3:** Standardized and unstandardized direct effects from the path model estimates

Direct effects	Estimate	Std Estimate	SE	*P* value
**Outcome: Respect and recognition**				
Staffing and Resources	0.20	0.24	0.02	<.001
Nurse Manager Leadership and Support	0.29	0.28	0.02	<.001
Foundations for Quality of Care	0.06	0.04	0.05	.249
Nurse Participation	0.46	0.40	0.03	<.001
Nurse-Physician Interaction	0.01	0.01	0.02	.681
**Outcome: Job enjoyment**				
Staffing and Resources	0.84	0.61	0.03	<.001
Nurse Manager Leadership and Support	0.48	0.28	0.03	<.001
Foundations for Quality of Care	0.00	0.00	0.07	.982
Nurse Participation	0.00	0.00	0.05	.949
Nurse-Physician Interaction	0.20	0.10	0.03	<.001
**Outcome: Missed nursing care**				
Staffing and Resources	-2.45	-0.87	0.08	<.001
Nurse Manager Leadership and Support	0.25	0.07	0.09	.008
Foundations for Quality of Care	0.52	0.10	0.23	.025
Nurse Participation	0.87	0.22	0.16	<.001
Nurse-Physician Interaction	-0.50	-0.12	0.09	<.001
**Outcome: Percent intent to stay**				
Staffing and Resources	18.50	0.44	0.01	<.001
Nurse Manager Leadership and Support	14.40	0.28	0.02	<.001
Foundations for Quality of Care	-11.30	-0.15	0.04	.004
Nurse Participation	-3.50	-0.06	0.03	.187
Nurse-Physician Interaction	5.60	0.09	0.02	<.001

Nurse manager leadership and support was significantly associated with all four outcomes. Notably, it had the strongest effect on respect and recognition (β = 0.28) and was also positively associated with retention (β = 0.28) and job enjoyment (β = 0.28). There was a small but statistically significant positive association with missed nursing care (β = 0.07), though the effect size was minimal. Foundations for quality of care was significantly associated with higher missed nursing care (β = 0.10) and lower retention (β = -0.15) but wasn't significantly related to respect and recognition or job enjoyment.

Nurse participation in hospital affairs was positively associated with respect and recognition (β = 0.40) and with missed nursing care (β = 0.22). However, it showed no significant association with retention or job enjoyment. Nurse-physician interaction had the weakest effects among all PES subscales. Although it was significantly associated with higher job enjoyment (β = 0.10), higher retention (0.09), and lower missed nursing care (β = -0.12), the effect sizes were small. It wasn't significantly related to respect and recognition. Overall, the model explained 30% of variance in retention, 47% in missed nursing care, 74% in respect and recognition, and 77% in job enjoyment. These results indicate that NWE most strongly explains variations in job enjoyment and perceptions of respect and recognition.

## DISCUSSION

This study sought to identify which domains of the NWE are most influential in shaping critical nurse outcomes, including retention, missed nursing care, job enjoyment, and feelings of respect and recognition. For nurse managers, these outcomes are central to building and sustaining an engaged and high-performing nursing workforce. These findings highlight that not all elements of the NWE contribute equally. Domains such as adequate staffing and resources and nurse manager leadership and support emerged as particularly influential, suggesting that targeted improvements in these areas may have the greatest impact on nurse retention, satisfaction, and quality of care delivered.

Although positive associations with PES subscales and favorable outcomes were observed, the analysis also revealed unexpected associations and patterns. For example, foundations for quality of care was negatively related to retention and nurse participation was related to an increase in missed nursing care. These patterns are consistent with interactive effects, indicating that the impact of any one domain may depend on the level of others. Additional investigation is warranted to disentangle these dependencies and test for interactive effects to better understand how the interplay among NWE domains shape nurse outcomes.

### Staffing and Resource Adequacy

Staffing and resource adequacy emerged as the most influential domain across all nurse outcomes. Units where nurses reported stronger staffing and resources had significantly higher levels of job satisfaction, retention, and feelings of respect and recognition, and fewer instances of missed nursing care. The strongest impact was on job enjoyment and missed nursing care. This finding is well supported in the literature; low nurse staffing is associated with a high prevalence of missed care, which in turn, has negative effects on patient and nurse outcomes.^10^ Further, Griffiths and colleagues demonstrated that understaffing negatively impacted quality of care and the well-being of nurses, increasing the risk of burnout.^11^ Although adequate staffing and resources are top priorities for nurse managers, given the current nursing shortage, this challenge may not be easily solved.^12^ Nurse managers need to be cognizant of the differences between the generations of nurses working in health care. Gen Z nurses, those currently up to age 27, are entering the workforce at an increasing rate. Although the fastest growing cohort of nurses, Gen Z nurses also had the highest turnover rate (27%) of any generation last year, compared with 21% of millennials, 13% of Gen X, and 19% of Baby Boomers.^13^ As a result, nurse managers are challenged to focus on retention of this group, whose primary expectations include purpose-drive work, physical and psychological safety, and meaningful investment in their professional growth.^13^,^14^ Where possible, providing flexibility and control over scheduling may help retain this growing cadre of nurses.^15^

### Nurse Manager Leadership and Support

Nurse manager leadership and support also emerged as a critical domain, especially in relation to nurses' feeling appreciated. Nurses who reported strong nurse manager leadership and support were also more satisfied with their role and planned to stay in their current position. The evidence is clear, nurse managers have long influenced nurses' job satisfaction, intent to stay, and well-being as well as improved patient quality and safety.^7^,^16^,^17^ Relational leadership styles are especially associated with significantly better nurse outcomes and NWE.^16^ Developing relational leadership style is critical for nurse managers given the impact on nurses' job satisfaction and intent to stay. Cummings and colleagues underscored the importance of formal mentorship programs connecting experienced leaders with emerging leaders.^16^ Leadership development, whether workshop-based or through ongoing mentorship, is vital for nurse managers.^18^

### Foundations for Quality of Care

The foundations for quality care subscale were linked to lower retention and more missed nursing care activities, counter to what was expected. This subscale includes items related to important structures and processes needed to support optimal nursing care and patient outcomes such as clinically competent nurses and high standards of nursing practice. It also contains two items that are no longer relevant to contemporary nursing practice; namely, use of nursing diagnosis and up-to-date care plans.^19^ This subscale should positively correlate with higher nurse job satisfaction and retention and fewer missed nursing care activities.^20^ Based on the simple regression analysis, interactive effects with other subscales are likely. In light of evidence linking environments supportive of quality care to better job satisfaction and fewer adverse nursing events, this interactive effect should be explored in future research.^14^,^21^

### Nurse-Physician Interaction

The results of this study demonstrated that nurse-physician interaction was significantly associated with higher job enjoyment, greater intent to stay, and lower missed nursing care. Strong nurse-physician interaction and teamwork is critical to quality patient care.^22^,^23^ Nurse managers should foster greater understanding of roles and expectations of nurses and physicians to promote positive nurse-physician interaction and collaboration.^22^ Team training may be one strategy to strengthen teamwork between nurses and physicians.

### Nurse Participation in Hospital Affairs

The nurse participation in hospital affairs subscale had the strongest effect in building respect and recognition among nurses. This subscale contains items that are focused on strong executive nursing leadership and nurses having a voice in the policies that govern nursing practice.^8^ The ability to exert control over one's practice is a powerful motivator and foundation for practicing at the top of one's professional license.^24^ Controlling nursing practice offers nurses the opportunity to eliminate nonnursing tasks, focus on the most meaningful aspects of their work, and enhance job satisfaction.^24^,^25^ Nurse managers should also encourage active participation in the organization's shared governance councils, which builds participation in nursing excellence initiatives, expression of concern regarding patient care issues, advocating for resources, and an overall enhanced NWE.^26^ Advocating for participation in hospital affairs, such as shared governance councils, builds professional well-being among nurses.

## RECOMMENDATIONS FOR NURSE MANAGERS

Based on our analysis, the primary drivers of nurse outcomes were staffing and resource adequacy and nurse manager leadership and support. Nurse-physician interaction and nurse participation in hospital affairs were secondary drivers of nurse outcomes. Interestingly, foundations for quality of care demonstrated unexpected significance; thus, it's also of importance as a driver. Therefore, to enhance nursing and patient outcomes, nurse managers need to engage in strategies that maximizes each subscale, given the subscale's impact on health care delivery. Evidence-based recommendations for nurse managers were culled from the literature and are reported by subscale in Table 4.

**TABLE 4:** Evidence-based recommendations for nurse managers by PES subscale

Subscale	Evidence-based strategies
Staffing and Resource Adequacy	Launch mentorship initiatives for early-career nurses^27^ Provide career advancement pathways and tuition support^28^ Develop and implement programs that promote the health and well-being of nurses^29^-^31^ Work with supply management to ensure adequate resources^32^ Implement innovative care delivery models^33^,^34^
Nurse Manager Leadership and Support	Offer professional development programs for nurse managers^35^ Encourage nurse leader rounding with their teams and patients^36^,^37^ Reimagine the nurse manager role to improve performance outcomes^38^,^39^ Create feedback loops and recognition systems^40^
Foundations for Quality of Care	Coach and mentor nurses on safety practices^41^,^42^ Promote evidence-based practice at the organization^43^ Lead discussions around unit performance metrics^44^
Nurse Physician Interaction	Promote team training to enhance skills, competency, and communication^45^ Implement interprofessional patient rounds^46^,^47^
Participation in Hospital Affairs	Identify opportunities for participation in shared governance activities^48^,^49^ Encourage evidence-based review of nursing procedures and policies^50^

## LIMITATIONS

Several limitations should be considered when interpreting these findings. First, although the path models allowed us to examine relationships among multiple NWE domains and nurse outcomes simultaneously, the cross-sectional design precludes causal inferences. As such, temporal ordering of NWE domains and nurse outcomes couldn't be established, limiting the ability to determine whether the observed associations reflect casual relationships in which specific aspects of the NWE lead to changes in nurse outcomes over time. Second, the sample was limited to hospitals participating in the NDNQI RN Survey, which may differ systematically from nonparticipating hospitals in organizational culture, NWE quality, or quality improvement engagement; therefore, the findings may not be generalizable to all hospital settings. Differences in these factors, which weren't measured as part of this study, could have influenced the observed relationships.

## GUIDANCE FOR NURSE RECRUITMENT AND RETENTION

This analysis underscored the critical importance of the NWE to nurse outcomes; specifically, respect and recognition, job enjoyment, missed nursing care, and intent to stay. Adequate staffing and competent nurse managers emerged as the strongest predictors for each of the outcomes. Nurse participation in decision-making and strong executive nurse leader presence were predictive of respect and recognition and missed nursing care. Collaborative nurse-physician relationships emerged as a weak determinant of job enjoyment, missed nursing care, and intent to stay.

Perhaps the most interesting finding was the negative effect of quality and safety on nurses' intent to stay in their same positions. The subscale held interactive effects with staffing and resources, reflecting additional burden to the work of nurses. The chief lesson from this study was that the individual domains of the NWE remain valid and provide guidance in recruiting and retaining qualified nurses—even Gen Z nurses.
